# AutoMamba: Efficient Autonomous Driving Segmentation Model with Mamba

**DOI:** 10.3390/s26072227

**Published:** 2026-04-03

**Authors:** Haoran Sun, Zhensong Li, Shiliang Zhu

**Affiliations:** 1 School of Information and Communication Engineering, The Center for Target Cognition Information Processing Science and Technology, Beijing Information Science and Technology University, Beijing 102206, China; 2023011135@bistu.edu.cn; 2 The State Key Laboratory of Information Engineering in Surveying, Mapping and Remote Sensing, Wuhan University, Wuhan 430079, China; 2024186190094@whu.edu.cn

**Keywords:** semantic segmentation, autonomous driving, state space models (SSM), mamba, linear complexity, real-time perception

## Abstract

Semantic segmentation for autonomous driving demands balancing high-fidelity perception with real-time latency. While Transformers achieve state-of-the-art results, their quadratic complexity bottlenecks high-resolution processing. State Space Models (SSMs) like Mamba offer linear complexity but often suffer from local detail loss and inefficient scanning strategies. We introduce AutoMamba, a tailored Hybrid-SSM architecture. We propose a Hybrid-SSM block incorporating Depthwise Convolutions to inject local spatial priors and a Stage-Adaptive Mixed-Scanning strategy. This strategy prioritizes horizontal context in early stages for road layouts while only activating vertical scanning in deep layers to preserve anisotropic structures like poles. Furthermore, we reveal that unlike Transformers, Mamba architectures require Auxiliary Supervision and Online Hard Example Mining (OHEM) to address “long-tail forgetting.” Experiments on Cityscapes and BDD100K under a training-from-scratch setting demonstrate AutoMamba’s superiority. Notably, AutoMamba-B0 achieves 67.79% mIoU on Cityscapes with 31.3% fewer FLOPs than SegFormer-B0. Moreover, while the larger SegFormer-B2 fails with Out-Of-Memory errors at 2048×2048 resolution, AutoMamba-B2 scales efficiently, validating its linear complexity advantage for next-generation perception systems.

## 1. Introduction

Autonomous driving systems rely heavily on the precise perception of complex road environments to ensure safety and navigation reliability. Among the core perception tasks, semantic segmentation plays a pivotal role by assigning a class label to every pixel in an image, thereby enabling the vehicle to distinguish traversable areas from critical obstacles and static infrastructure. Given the safety-critical nature of autonomous driving, segmentation models face a rigorous dual requirement: they must achieve high precision to minimize perceptual errors and maintain low latency for real-time decision-making on resource-constrained edge devices.

In recent years, the landscape of semantic segmentation has been dominated by two major paradigms: Convolutional Neural Networks (CNNs) and Vision Transformers (ViTs) [1,2,3,4,5,6]. While CNNs efficiently capture local features, they often struggle with long-range dependencies due to their limited receptive fields. Conversely, although ViTs successfully model global context via Self-Attention, they suffer from prohibitive computational costs on high-resolution driving imagery. Although state-of-the-art methods like SegFormer attempt to mitigate this by optimizing sequence length [7], they remain fundamentally bound by the quadratic complexity of the attention mechanism. As image resolution increases to detect distant obstacles, this computational bottleneck persists, making Transformer-based models challenging to deploy in resource-constrained, real-time autonomous driving systems.

To overcome this quadratic barrier, State Space Models (SSMs), particularly the Mamba architecture [8], have recently attracted attention for their ability to model long sequences with linear complexity. Mamba relies on a Selective Scan Mechanism (S6) that compresses context into a hidden state, allowing for efficient parallel training and extremely fast inference. First devised for 1D sequence modeling in natural language processing (NLP), Mamba has since been extended to the visual domain [9]. By treating images as flattened sequences, Mamba offers a theoretical path to achieving the global receptive field of Transformers without their heavy computational cost.

However, directly applying vanilla Mamba to the complex 2D scenes of autonomous driving presents unique challenges. First, standard 1D scanning may lose spatial continuity, particularly for objects with extreme aspect ratios common in street scenes—such as tall, thin utility poles—which span long vertical distances but short horizontal ones. Second, while Mamba excels at global context, it may underperform in capturing fine-grained local textures compared to convolutions.

**Figure 1:**
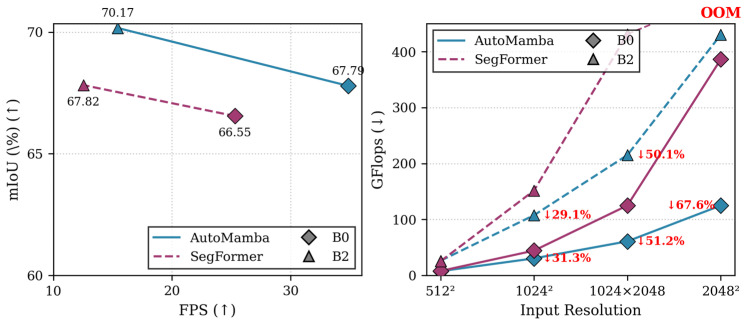
Efficiency and Scalability Analysis on Cityscapes. (**left**) Accuracy vs. Speed Trade-off: Comparison of mIoU (↑ indicates higher is better) and FPS (↑) under the “training from scratch” setting. Our AutoMamba (solid blue lines) consistently outperforms the state-of-the-art SegFormer (dashed pink lines), achieving higher segmentation accuracy with faster inference speed across both B0 and B2 variants. (**right**) Complexity Scaling: Comparison of GFLOPs (↓ indicates lower is better) growth with increasing input resolution. Thanks to the linear complexity $O\left(N\right)$ of the Mamba architecture, AutoMamba scales efficiently, achieving a computational reduction of up to 67.6% (denoted by the red downward arrows and percentages) at high resolutions compared to the quadratic $O\left(N^{2}\right)$ Transformer baseline. Notably, SegFormer-B2 fails with Out-Of-Memory (highlighted as red OOM) errors at 2048 × 2048 resolution on a 24 GB GPU, whereas AutoMamba remains computationally viable.

In summary, existing perception paradigms present a critical dilemma for autonomous driving. While industry-standard CNNs (e.g., DeepLab series) are hardware-friendly, their reliance on dilated convolutions to expand receptive fields often introduces gridding artifacts and inherently lacks true global scene understanding. Conversely, ViTs suffer from prohibitive quadratic computational bottlenecks, leading to severe latency and Out-Of-Memory (OOM) errors on vehicle edge devices when processing high-resolution imagery, as illustrated in the right panel of Figure 1. Although vanilla SSMs offer a theoretical linear complexity alternative, their naive 1D scanning violently disrupts 2D spatial continuity. In safety-critical driving scenarios, these geometric and computational flaws are catastrophic: they lead to the fragmentation of objects with extreme aspect ratios and the attenuation of essential local textures, potentially causing fatal misclassifications or missed detections of life-threatening obstacles. Therefore, it is absolutely crucial to design a perception architecture that breaks this compromise—one that synergizes the local precision of CNNs, the global awareness of ViTs, and the real-time efficiency of SSMs without sacrificing geometric integrity.

Driven by this vital need, in this paper, we propose a novel Mamba-based Semantic Segmentation Network designed to surpass SegFormer in both speed and accuracy. We introduce a Mixed-Direction Scanning strategy that enhances the memory capacity for vertically elongated objects, ensuring robust segmentation of thin infrastructure like poles. Furthermore, we construct a Hybrid SSM Module that explicitly embeds a 3×3 Depthwise Convolution within each Mamba State Space Module. This hybrid design enforces a complementarity between global context and local details, while the convolution aids in memory rearrangement to stabilize the feature extraction process.

Our contributions are summarized as follows:

- Linear Complexity Architecture: We propose a Mamba-based hybrid encoder architecture for semantic segmentation that effectively breaks the quadratic complexity bottleneck of SegFormer, offering a superior trade-off between computational efficiency and segmentation quality. Compared with the vanilla Mamba baseline, this hybrid design remedies the defects of local detail loss and inefficient spatial modeling in pure Mamba architectures, and yields a remarkable enhancement in the model’s capability to capture fine-grained features and anisotropic structures in complex driving scenes with only a minimal increase in parameters and computational overhead.
- Context-Aware Innovation: We introduce a Mixed-Direction Scanning Mamba to specifically address the challenge of segmenting objects with extreme aspect ratios (e.g., utility poles), and a Hybrid SSM Block incorporating Depthwise Convolution to synergize global context modeling with local feature refinement.
- Empirical Superiority: We conduct extensive experiments on the Cityscapes and BDD100K datasets, with ablation studies on Cityscapes verifying remarkable performance gains over the vanilla Mamba baseline. As comprehensively depicted in Figure 1, our method achieves 67.28% mIoU and 74.99% Accuracy, outperforming SegFormer-B0 by 0.74 points while reducing computational cost by 31.3% (measured in FLOPs) and improving inference speed to 34.85 FPS. Specifically, the full-configured AutoMamba achieves a substantial 9.46 percentage point mIoU gain over the vanilla Mamba baseline in ablation experiments, and this remarkable performance boost is attained with only minimal increments in parameters (from 3.908 M to 3.918 M) and GFLOPs (from 30.381 to 30.455). This fully validates the synergetic and highly efficient design of our proposed Hybrid-SSM block and Stage-Adaptive Mixed-Scanning strategy. Notably, we deliver superior performance on the safety-critical classes essential for autonomous driving scenarios, validating the robustness of our approach and its distinct advantages over the vanilla Mamba baseline for the recognition of key traffic targets.

## 2. Related Work

### 2.1. Evolution of Semantic Segmentation: CNNs and ViTs

Semantic segmentation has historically advanced through two dominant paradigms. CNNs established the foundation, with FCNs [10] pioneering end-to-end dense prediction. Subsequent architectures like DeepLab [11,12,13,14] and PSPNet [15] utilized dilated convolutions and pyramid pooling to expand receptive fields. However, CNNs remain constrained by their inherent local inductive bias, struggling to capture the long-range dependencies required to interpret complex, large-scale driving scenes.

ViTs transcended this locality via the Self-Attention mechanism. While SETR [16] proved the feasibility of ViTs for segmentation, architectures like Swin Transformer [17,18] optimized efficiency through window-based attention. SegFormer [7] further advanced this by reducing complexity to $O\left(N^{2}/R\right)$ via sequence reduction. Nevertheless, it retains the fundamental quadratic bottleneck of self-attention. As autonomous driving demands higher resolution imagery for distant object detection, this quadratic scaling leads to prohibitive computational costs, hindering deployment on resource-constrained edge platforms.

### 2.2. SSMs and Mamba

To address the quadratic inefficiency, research has revisited State Space Models (SSMs) [19,20,21,22], which offer the linear complexity $O\left(N\right)$ of Recurrent Neural Networks (RNNs) [23,24] without their training parallelization constraints. Early variants like S4 [20] combined continuous-state formulations with structured parameterizations (e.g., HiPPO matrices [19,25,26,27]) to model long-range dependencies efficiently.

Building on S4, Mamba introduced the Selective Scan Mechanism (S6) [8]. Unlike S4, S6 makes the system parameters (B,C,Δ) input-dependent, allowing the model to selectively propagate or forget information based on context. To enable efficient training despite this time-varying nature, Mamba implements a hardware-aware parallel scan algorithm, achieving Transformer-level performance with linear inference cost.

### 2.3. Visual State Space Models

Adapting Mamba to 2D vision requires serializing image data. Vision Mamba (Vim) [28] flattens images into sequences and employs bidirectional horizontal scanning. While efficient, this approach often disrupts the semantic continuity of vertically elongated objects (e.g., poles) typical in street scenes. VMamba [29] addresses this by introducing a Cross-Scan Module (CSM), which traverses features bidirectionally along both horizontal and vertical axes across the entire network.

However, significant limitations remain for autonomous driving. First, VMamba’s uniform application of 4-way scanning essentially quadruples the SSM computational load, creating a new latency bottleneck. Second, treating all network stages identically ignores the hierarchical nature of visual features—where early stages require local texture coherence and deep stages require global semantic context.

### 2.4. Mamba in Dense Prediction Tasks

Following the theoretical success of State Space Models, recent research has rapidly expanded Mamba’s application to dense prediction tasks, exploring various structural modifications to adapt 1D sequence modeling for high-resolution spatial features [30,31,32,33,34,35,36].

A dominant paradigm involves hybridizing Mamba with specialized convolutional modules to enhance spatial feature extraction. SegMamba [31] represents a pioneering effort in this direction, designing a Gated Spatial Convolution (GSC) [37] module placed before the SSM block. This module utilizes element-wise gating mechanisms to selectively emphasize relevant spatial features before sequence modeling. Similarly, VM-UNet++ [33,34] adapts nested dense skip connections to Vision Mamba to facilitate feature reuse across scales, while Deformable Mamba [32] integrates deformable convolutions to handle geometric irregularities. Other approaches, such as MFMamba [35], employ dual-branch architectures that fuse features from a CNN branch and a Mamba branch to balance local and global contexts.

While these architectures demonstrate the feasibility of Mamba for dense prediction, their structural designs present critical inefficiencies when applied to the stringent latency constraints of autonomous driving. Although the GSC used in SegMamba effectively enhances feature representation, it relies on multi-branch structures and element-wise multiplication. On edge computing hardware typical of autonomous vehicles, these memory-bound operations often induce higher latency compared to streamlined operations. For real-time applications, the computational cost of complex gating outweighs the marginal gain in spatial precision. Existing methods, including the Tri-orientated Mamba (ToM) in SegMamba or generic cross-scanning modules in other works, typically apply multi-directional scanning uniformly across all network stages. This ignores the hierarchical nature of visual features: in early high-resolution stages, features are dominated by local texture where simple scanning suffices. Applying complex multi-directional scanning here incurs computational redundancy without proportional semantic gain. In contrast, broader dense prediction domains have successfully leveraged stage-aware and locally enhanced designs. For instance, in Optical Remote Sensing Image (ORSI) salient object detection, progressive interaction and saliency-guided enhancement mechanisms have proven highly effective in capturing irregular topological structures [38]. Such progressive and locally guided philosophies provide conceptual inspiration for rethinking our architectural design.

As summarized in Table 1, existing paradigms exhibit inherent limitations in balancing computational efficiency and global contextual awareness. Consequently, there remains a crucial need for a streamlined architecture that optimizes the scanning strategy to enhance structural feature extraction while strictly guaranteeing real-time inference speed.

## 3. Methods

In this section, we formulate AutoMamba, a hierarchical semantic segmentation architecture designed to mitigate the quadratic complexity bottleneck of Transformers while preserving high-fidelity modeling of complex driving scenes. We first delineate the macroscopic pipeline, followed by a rigorous exposition of our three core contributions: the Hybrid SSM Block with Local Prior Injection, the Stage-Adaptive Mixed-Direction Scanning Strategy, and the Deep Supervision Training Objective.

### 3.1. Overall Architecture

**Figure 2:**
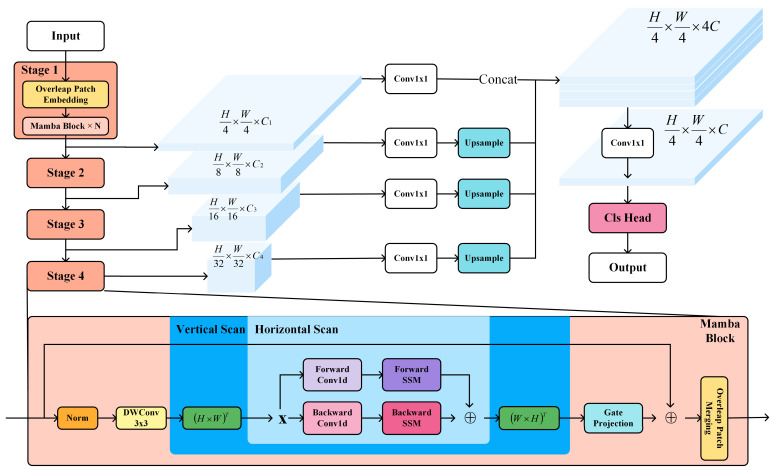
Overall architecture of the proposed AutoMamba framework for semantic segmentation. The encoder consists of four hierarchical stages with Overlap Patch Embedding and stacked Bidirectional Mamba Encoder Layers, outputting multi-scale features (C1–C4) at different resolutions (H/4 to H/32) with progressively increasing channels (64, 128, 256, 512). The decoder employs an all-MLP head with 1 × 1 convolutions, bilinear upsampling, and feature concatenation to generate the final segmentation map. The bottom panel illustrates the detailed structure of a Mamba Block, which integrates 3 × 3 depthwise convolution (DWConv) for local feature extraction, bidirectional State Space Models (SSM) for long-range dependency modeling with configurable scan directions (horizontal/vertical), and gated projection for adaptive feature selection.

To ensure a rigorous evaluation of our core architectural innovations—isolating the contribution of the state space model from macroscopic structural variances—we align our topology with the established hierarchical Encoder–Decoder paradigm. The overall architecture of our proposed model is depicted in Figure 2.

Encoder (Hierarchical Mamba): The encoder comprises four stages with downsampling ratios of 1/4, 1/8, 1/16, 1/32. In contrast to standard Vision Transformers (e.g., SegFormer) which employ Multi-Head Self-Attention (MHSA) with quadratic complexity $O\left(N^{2}\right)$, we construct each stage using our proposed Hybrid Mamba Blocks. This design maintains linear complexity $O\left(N\right)$ with respect to the sequence length N=H×W. The channel dimensions for the four stages are scaled as $C_{i}\in 32,\;64,\;160,\;256$ to ensure parameter parity with SegFormer-B0.

Decoder (MLP Head): To validate the representational strength of the encoder features, we utilize a lightweight All-MLP Decoder. Multi-scale features are upsampled to a unified resolution (H/4×W/4), concatenated, and fused via a 1×1 convolution to project the final segmentation map $M\in R^{H\times W\times N_{cls}}$, where $N_{cls}$ denotes the number of semantic classes.

### 3.2. Hybrid SSM Block with Local Prior Injection

Standard Vision Mamba architectures serialize images into flattened 1D sequences, relying exclusively on the State Space Model (SSM) to model spatial dependencies. However, this serialization disrupts the intrinsic 2D local structure, rendering the model susceptible to “forgetting” high-frequency details critical for small targets (e.g., distant pedestrians). To address this, we introduce the Hybrid SSM Block, which injects a local spatial prior before sequence modeling.

Let $X_{in}\in R^{H\times W\times C}$ denote the input feature map. We first apply Layer Normalization followed by a DWConv [39]. This operation functions as a logical Local Prior, sharpening object boundaries and capturing immediate neighborhood correlations without imposing significant computational overhead:

(1) $$X_{\mathrm{local}}={\mathrm{DWConv}}_{3\times 3}\left(\mathrm{Norm}\left(X_{\mathrm{in}}\right)\right)$$

Subsequently, $X_{local}$ is flattened into tokens $T\in R^{L\times C}$ (where L=HW) and processed by the Mamba module. The core of this module is the Selective State Space Model (S6), which projects a 1-dimensional input sequence $x_{t}\in R$ into an implicit N-dimensional latent state $h_{t}\in R^{N}$ before projecting it back to an output sequence $y_{t}\in R$. This process is governed by a continuous-time linear ordinary differential equation (ODE):

(2) $$h_{t}^{\prime }=Ah_{t}+Bx_{t}$$

(3) $$y_{t}=Ch_{t}$$

Here, the matrices are rigorously defined as follows: $A\in R^{N\times N}$ is the state evolution matrix, which captures the historical context and governs how the hidden state memory transitions over time; $B\in R^{N\times 1}$ is the input projection matrix, mapping the current observation into the high-dimensional latent space; and $C\in R^{1\times N}$ is the output projection matrix, mapping the latent state back to the target output dimension.

To apply this continuous-time dynamical system to discrete token sequences (e.g., flattened image patches), the system must be discretized. We employ the Zero-Order Hold (ZOH) discretization rule. By introducing a timescale parameter Δ—which controls the resolution of the continuous dynamics and is made input-dependent to selectively filter information—the continuous parameters A and B are analytically integrated into their discrete counterparts, denoted as $\bar{A}$ and $\bar{B}$:

(4) $$\bar{A}=\exp\left(\Delta A\right)$$

(5) $$\bar{B}={\left(\Delta A\right)}^{-1}\left(\exp\left(\Delta A\right)-I\right)\cdot \Delta B$$

Using these discrete matrices, the state space model transforms into an efficient linear recurrence relation, enabling sequence processing:

(6) $$h_{t}=\bar{A}h_{t-1}+\bar{B}x_{t}$$

(7) $$y_{t}=Ch_{t}$$

By explicitly modeling this rigorous discretization, S6 maintains the theoretical properties of continuous long-range memory while allowing for hardware-aware parallelization during training.

To further enhance channel interaction and introduce non-linearity, we append a Gated MLP following the bidirectional SSM fusion. Let $Y_{out}$ be the sum of bidirectional streams, the final block output $X_{out}$ is computed as:

(8) $$G=\mathrm{Linear}D\to 2D\left(Y_{\mathrm{out}}\right)X_{\mathrm{out}}=X_{\mathrm{in}}+\mathrm{Dropout}\left(G\left[0:D\right]⊙\mathrm{SiLU}\left(G\left[D:2D\right]\right)\right)$$

⊙ denotes element-wise multiplication, and G is split into two halves along the channel dimension.

### 3.3. Stage-Adaptive Mixed-Direction Scanning Strategy

A critical limitation of existing Vision Mamba architectures is the reliance on row-major (horizontal) scanning. In autonomous driving scenarios, numerous critical obstacles—such as utility poles, traffic signs, and street lamps—exhibit extreme aspect ratios (i.e., they are vertically elongated). A horizontal scan slices these objects into disjointed segments separated by extensive background intervals, weakening semantic correlation. To resolve this geometric mismatch, we propose a Stage-Adaptive Mixed-Direction Scanning Strategy.

- Horizontal Dominance (Stages 1–2): In early high-resolution stages, local texture modeling is paramount. We employ standard bidirectional horizontal scanning to capture the general scene layout.
- Vertical Introduction (Stage 3): As the receptive field expands, we transition the scanning logic. For the latter blocks of Stage 3, we integrate Vertical Bidirectional Scanning.
- Vertical Dominance (Stage 4): In the deepest stage, we exclusively employ vertical scanning to capture global vertical context (e.g., sky–road relationships and building height).

Mathematically, for a vertical scan, the feature map X is transposed prior to flattening: $T_{vert}=\mathrm{Flatten}\left(X^{⊤}\right)$. The bidirectional output $Y_{out}$ is the summation of the forward ($\overset{\to }{S}$) and backward ($\overset{\leftarrow }{S}$) scans:

(9) $$Y_{\mathrm{out}}={\mathrm{SSM}}_{\mathrm{fwd}}\left(T\right)+\mathrm{Flip}\left({\mathrm{SSM}}_{\mathrm{bwd}}\left(\mathrm{Flip}\left(T\right)\right)\right)$$

We term this strategy ‘Stage-Adaptive’ because the scanning direction is explicitly designed to align with the evolving intrinsic properties of the feature maps at each network stage—transitioning from local texture modeling in shallow layers to global semantic structure modeling in deep layers. This strategy establishes a “vertical memory tunnel,” ensuring that the semantic features of a pole’s apex effectively communicate with its base, thereby recovering structural integrity.

### 3.4. Training Objective with Auxiliary Supervision

Due to the recurrent nature of SSMs, the hidden state is susceptible to spatial memory decay when processing extremely long flattened 2D sequences—an architectural vulnerability we specifically refer to as “spatial long-tail forgetting.” This vanishing gradient issue implies that fine-grained features of targets appearing early in the scanning sequence may be lost by the time the sequence terminates. In autonomous driving datasets, this physical sequence forgetting severely exacerbates the statistical class imbalance problem; subtle features of rare or small targets (e.g., pedestrians, traffic signs) are easily overwritten by the continuous accumulation of dominant background classes (e.g., road, sky). To mitigate this dual challenge of spatial decay and class bias, we introduce Auxiliary Supervision at Stage 3.

We append an auxiliary MLP head to the output of Stage 3. The total objective function $L_{total}$ is the weighted summation of the primary loss $L_{main}$ and the auxiliary loss $L_{aux}$:

(10) $$L_{\mathrm{total}}=L_{\mathrm{main}}\left(P_{\mathrm{final}},Y_{\mathrm{gt}}\right)+\lambda \cdot L_{\mathrm{aux}}\left(P_{\mathrm{stage}3},Y_{\mathrm{gt}}\right)$$

Here, λ is empirically set to 0.2 to balance the loss components. Moreover, we employ the Online Hard Example Mining (OHEM) Cross-Entropy Loss for the primary segmentation head. This strategy imposes a severe penalty on misclassified hard pixels, thereby compelling the Mamba latent states to preserve discriminative features critical for recognizing small, difficult targets.

## 4. Experiments

### 4.1. Experimental Setup

We evaluate our proposed AutoMamba on two standard autonomous driving benchmarks: Cityscapes [40] and BDD100K [41]. Cityscapes consist of 5000 fine-annotated images (1024×2048) capturing urban street scenes, split into 2975 for training and 500 for validation. To further assess robustness under diverse weather and lighting conditions, we also employ the large-scale BDD100K dataset, which comprises 7000 training images and 1000 validation images. All experiments are conducted using the MMSegmentation framework (version 1.2.2) based on PyTorch (version 2.1.0+cu121) on a single NVIDIA RTX 4090 GPU (NVIDIA Corp., Santa Clara, CA, USA) hosted on a cloud server provided by AutoDL (Nanjing, China).

Network Configurations: Regarding the model variants evaluated, the Stage-Adaptive scanning strategy adapts dynamically to the depth of the model architecture. Specifically, we adhere to the rule where vertical scanning is activated only after the first two blocks in Stage 3. For the lightweight AutoMamba-B0, which utilizes a block configuration of $\left[2,\;2,\;2,\;2\right]$, Stage 3 contains exactly two blocks; consequently, Stage 3 in B0 remains entirely horizontal, with vertical scanning reserved exclusively for Stage 4. In contrast, for the deeper AutoMamba-B2 (configuration $\left[3,\;4,\;6,\;3\right]$), Stage 3 comprises six blocks. Therefore, the first two blocks perform horizontal scanning to maintain feature consistency, while the subsequent four blocks transition to vertical scanning, thereby enabling a progressive and seamless expansion of the vertical receptive field. The models are optimized using AdamW with an initial learning rate of $6\times 10^{-5}$ and a weight decay of 0.01, following a polynomial learning rate schedule with a power of 1.0. During training, we apply standard data augmentations, including random scaling (0.5 to 2.0), flipping, and cropping to a resolution of 1024×1024.

Crucially, to ensure a scientifically rigorous comparison, all models, including the SegFormer baseline, are trained entirely from scratch without ImageNet pre-training. We deliberately adopt this strictly controlled “clean-slate” paradigm for several key reasons. First, it isolates the architectural contributions, guaranteeing that performance gains are driven intrinsically by our Hybrid-SSM and Stage-Adaptive Scanning designs rather than the confounding effects of massive external data. Second, generic ImageNet pre-training introduces a significant domain mismatch; it lacks the specific geometric priors crucial for autonomous driving, such as street scenes, utility poles, and traffic signs. Third, in real-world industrial deployment, massive pre-training overhead is often prohibitive for rapid iteration, and many pipelines prefer end-to-end training directly from raw, domain-specific sensor data. By maintaining this scratch-training protocol, we directly evaluate the models’ intrinsic data efficiency and optimization stability. Specifically, this setting demonstrates that our hybrid design (DWConv + SSM) provides a strong inductive bias for local-global modeling, effectively reducing the reliance on external pre-training. For the main comparison, models are trained for 160 K iterations with a batch size of 8 (for B0) or 2 (for B2). Ablation studies are conducted on a shortened schedule of 80 K iterations to efficiently verify the contribution of each architectural module.

### 4.2. Comparison with State-of-the-Art

We benchmark AutoMamba against SegFormer, a representative Transformer-based architecture, under identical training constraints. The quantitative results on Cityscapes and BDD100K are reported in Table 2, per-class performance is detailed in Table 3, and computational complexity is analyzed in Table 4.

Accuracy and Efficiency Trade-off: On the Cityscapes validation set, AutoMamba-B0 achieves a mean IoU (mIoU) of 67.79%, surpassing the SegFormer-B0 baseline (66.55%) by a margin of 1.24%. Notably, this accuracy gain is accompanied by a significant improvement in inference speed, with AutoMamba-B0 reaching 34.85 FPS compared to SegFormer-B0’s 25.31 FPS. This trend scales effectively with model capacity; the larger AutoMamba-B2 variant reaches 70.17% mIoU, significantly outperforming SegFormer-B2 (67.82%) by 2.35%.

Computational Scalability: As detailed in Table 4, the proposed architecture exhibits remarkable efficiency, particularly at higher resolutions. At a standard input size of 1024×1024, AutoMamba-B0 requires only 30.45 GFLOPs, representing a 31.3% reduction in computational cost compared to SegFormer-B0 (44.30 GFLOPs). The advantage of linear complexity becomes pronounced at high resolutions: at 2048×2048, SegFormer-B2 fails due to OOM errors, whereas AutoMamba-B2 remains computationally viable with 430.08 GFLOPs.

Per-Class Analysis: Table 3 provides a granular look at class-wise performance. Consistent with our design goal of enhancing vertical context, AutoMamba demonstrates distinct improvements in vertically elongated classes. For instance, AutoMamba-B0 improves the IoU for Poles by +2.78% (49.99 → 52.77%) and Trucks by +12.78% (55.23% → 68.01%) compared to SegFormer-B0. Similarly, AutoMamba-B2 shows robust gains in dynamic classes, such as Riders (+7.01%) and Trains (+12.19%), validating the model’s ability to maintain structural integrity for complex objects.

Generalization on BDD100K: The robustness of our approach is further confirmed on the diverse BDD100K dataset. AutoMamba-B0 achieves 46.86% mIoU, consistently outperforming the baseline (44.86%) by 2.0%, demonstrating strong generalization capabilities across varying weather and lighting conditions.

### 4.3. Analysis of Optimization Dynamics

A key finding of this study is the distinct response of Mamba-based and Transformer-based architectures to advanced supervision strategies, specifically Auxiliary Heads (Aux) and Online Hard Example Mining (OHEM). As detailed in Table 5, we observe that applying Aux + OHEM to SegFormer-B0 results in a performance degradation (59.53% → 58.16% mIoU). We attribute this to the global attention mechanism of Transformers, which inherently facilitates gradient flow; enforcing excessive focus on hard examples via OHEM may disrupt the attention map optimization in the absence of pre-trained weights.

In contrast, AutoMamba-B0 benefits significantly from this strategy, achieving a +0.97% improvement (58.65% → 59.62% mIoU). This empirical evidence supports our theoretical analysis: the recursive state-space mechanism is prone to “long-tail forgetting,” where information regarding small or rare targets decays over long sequences. The auxiliary supervision acts as a critical regularizer, forcing the SSM to retain fine-grained features in intermediate states, thereby validating the necessity of deep supervision for Mamba-based segmentation models.

### 4.4. Ablation Study

To investigate the effectiveness of our architectural components, we conducted a progressive ablation study on Cityscapes (80 K iterations), as summarized in Table 6.

To ensure a rigorous and reproducible evaluation, we first explicitly define the “Pure Mamba” baseline. This baseline configuration is constructed by stripping our proposed Hybrid-SSM block of its 3 × 3 Depthwise Convolution (DWConv) and completely disabling the Stage-Adaptive Mixed-Scanning strategy. Consequently, the “Pure Mamba” model processes the flattened 2D feature maps utilizing only standard bidirectional horizontal scanning across all four network stages. This configuration mirrors the fundamental 1D sequence modeling approach of vanilla Vision Mamba architectures, serving as a strict control variable.

As shown in Table 6, this “Pure Mamba” baseline achieves a suboptimal mIoU of 49.19%. This verifies our hypothesis that relying solely on 1D horizontal sequence modeling is insufficient for capturing the complex 2D spatial structures inherent in driving scenes. The injection of local spatial priors via DWConv significantly boosts performance to 55.18% (+5.99%), confirming that explicit local modeling is essential to prevent the loss of high-frequency details before sequence processing. Furthermore, integrating our Stage-Adaptive Mixed-Scanning strategy (activating vertical scanning in Stage 4) yields a substantial improvement on its own, raising the mIoU to 57.74%. This highlights the critical role of vertical context in preserving the structural integrity of anisotropic objects. The final configuration, which synergizes both DWConv and Mixed-Scanning, achieves the highest performance of 58.65%, demonstrating the complementarity of local spatial priors and global vertical context.

## 5. Discussion

In this section, we interpret the internal feature aggregation mechanisms of AutoMamba through Effective Receptive Field (ERF) visualization and analyze how these mechanisms translate into qualitative segmentation improvements.

### 5.1. Analysis of Effective Receptive Field Evolution

To generate the Effective Receptive Field (ERF) maps, we strictly adhered to a rigorous gradient backpropagation methodology. Let $X^{\left(i\right)}\in R^{3\times H_{in}\times W_{in}}$ denote the i-th input image, and $Y^{\left(i\right)}\in R^{C\times H_{out}\times W_{out}}$ represent the corresponding output feature map from a given network stage. We isolate the central spatial position $\left(u,v\right)=\left(H_{out}/2,W_{out}/2\right)$ and define the target response signal $y_{c}^{\left(i\right)}$ by summing the activations across all C channels:

(11) $$y_{c}^{\left(i\right)}=\sum_{j=1}^{C}Y_{j,u,v}^{\left(i\right)}$$

We then backpropagate this scalar signal to the input image plane to obtain the gradient map $G^{\left(i\right)}=\partial y_{c}^{\left(i\right)}/\partial X^{\left(i\right)}$. To quantify the spatial influence of each input pixel $\left(x,y\right)$, we compute the Root Mean Square (RMS) of the gradients across the three color channels:

(12) $$E^{\left(i\right)}\left(x,y\right)=\sqrt{\frac{1}{3}\sum_{c=1}^{3}{\left(G_{c,x,y}^{\left(i\right)}\right)}^{2}}$$

To ensure statistical robustness and eliminate image-specific biases, these spatial influence maps are accumulated and averaged over N=100 randomly sampled images (resized to 1024 × 1024) from the Cityscapes validation set. Finally, to effectively visualize long-range dependencies that might otherwise be visually suppressed, a logarithmic transformation is applied to the averaged map:

(13) $$ERF=\log_{10}\left(\frac{1}{N}\sum_{i=1}^{N}E^{\left(i\right)}+\epsilon \right)$$

where $\epsilon =10^{-10}$ is a small constant to prevent numerical instability. The resulting ERF map is then normalized to a $\left[0,1\right]$ range for pseudo-color visualization.

**Figure 3:**
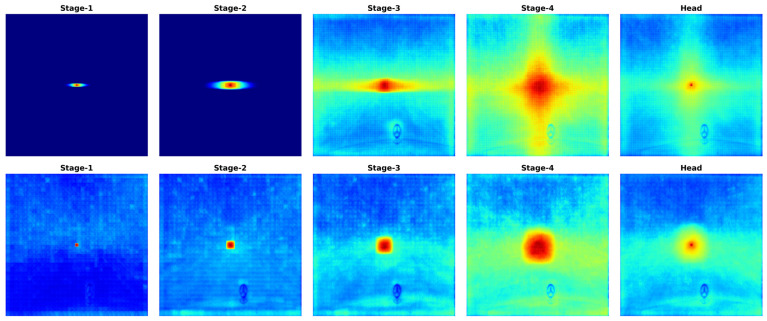
Visualization of the Effective Receptive Field (ERF) for the central pixel across different stages. Top row (AutoMamba): The ERF evolves from a horizontally concentrated pattern (Stages 1–2) to a global field (Stage 3) and finally forms a cross-shaped topology with vertical extension (Stage 4). Bottom row (SegFormer): The ERF remains isotropic and Gaussian-distributed throughout all stages. Note: The colors in the heatmaps represent the intensity of the ERF. Warmer colors (e.g., red) indicate regions with higher contribution weights to the central feature, whereas cooler colors (e.g., blue) represent lower or negligible impact.

A comparative analysis with the SegFormer baseline reveals a clear evolution in feature integration logic. In the initial high-resolution stages (Stages 1 and 2), AutoMamba, as depicted in Figure 3, exhibits a highly concentrated, horizontally elongated ERF. This distribution confirms that the model prioritizes lateral context in shallow layers, effectively suppressing vertical noise while capturing continuous road layouts. The tight concentration of the response field also verifies the role of DWConv in enforcing local spatial locality, thereby preventing the feature dilution often observed in pure SSM-based architectures. In contrast, SegFormer displays a diffuse, isotropic attention pattern lacking directional selectivity.

A critical transition is observed in Stage 3, where the ERF undergoes a significant expansion, covering a substantial portion of the image. This indicates that despite the local constraints imposed in early stages, the Mamba core successfully models long-range dependencies once the feature abstraction level increases. This global expansion provides the necessary semantic context for subsequent fine-grained structural modeling. In the final stage (Stage 4), the ERF of AutoMamba evolves into a distinctive cross-shaped topology, characterized by a prominent vertical extension intersecting with the horizontal field. This phenomenon is a direct consequence of the Stage-Adaptive Mixed-Scanning strategy, where vertical scanning is exclusively activated in the deepest layer. This orthogonal structural integration enables the model to simultaneously perceive the horizon and vertical height. Conversely, SegFormer’s ERF remains Gaussian-distributed and isotropic. The significantly larger and structurally aligned ERF of AutoMamba in Stage 4 explains its superior capability in handling anisotropic objects without the quadratic computational cost associated with Transformers.

### 5.2. Qualitative Performance Analysis

**Figure 4:**
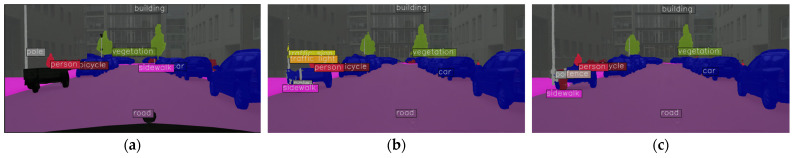
Qualitative comparison on Cityscapes. (**a**) Ground Truth. (**b**) SegFormer-B2: Fails to capture the vertical pole on the left, resulting in fragmentation and misclassification as “traffic sign/light.” (**c**) AutoMamba-B2 (Ours): Successfully segments the complete pole structure, demonstrating the effectiveness of the Stage-Adaptive Mixed-Scanning strategy in preserving vertical context.

The theoretical advantages observed in the ERF visualizations are corroborated by the qualitative segmentation results on the Cityscapes validation set (Figure 4). As hypothesized from the Stage 4 vertical ERF expansion, AutoMamba demonstrates superior performance in preserving the integrity of vertically elongated structures.

A striking example is provided in the comparison of the utility pole on the left side of the scene. As shown in Figure 4b, the SegFormer-B2 baseline fails to model the global vertical context of the pole. Consequently, it not only fragments the continuous structure but also suffers from semantic ambiguity, yielding false positive predictions (misclassifying sections of the pole as “traffic sign” and “traffic light”). In contrast, AutoMamba-B2 (Figure 4c) effectively mitigates this issue. Leveraging the vertical scanning mechanism in the deep stages, our model successfully integrates context from the ground to the top of the pole, generating a coherent and continuous mask that closely matches the Ground Truth.

Furthermore, for complex object boundaries such as the person and bicycle in the center-left region, AutoMamba yields sharper delineations with fewer false negatives compared to the baseline. This improvement is attributed to the Hybrid-SSM design, where the local inductive bias retained via DWConv prevents high-frequency details from being washed out during global aggregation. Additionally, the application of OHEM helps stabilize the learning of these thinner, less frequent structures, ensuring they are not overwhelmed by the dominant road and building classes during optimization.

## 6. Conclusions

In this work, we presented AutoMamba, a novel framework that successfully adapts the linear complexity Mamba architecture to the constraints of autonomous driving segmentation. By analyzing the limitations of existing Vision Mamba approaches, we identified two critical gaps: the lack of local spatial priors and the inefficiency of isotropic scanning strategies. We addressed these via a Hybrid-SSM design and a geometrically aware Stage-Adaptive Mixed-Scanning strategy. Our results lead to three key conclusions:

1. Geometric Adaptation: Aligning the scanning direction with the scene’s inherent geometry (i.e., introducing vertical scanning only in deep layers) significantly enhances the segmentation of anisotropic structures like poles and signs, outperforming generic scanning methods with lower computational cost.
2. Efficiency-Scalability Superiority: AutoMamba demonstrates a decisive advantage in high-resolution processing. Unlike Transformers which suffer from quadratic complexity and OOM failures, our method maintains linear scalability, making it an ideal candidate for next-generation high-definition perception systems.
3. Optimization Insight: We uncovered that Mamba architectures are more sensitive to long-tail class forgetting than Transformers. We empirically verified that strong supervision strategies (Auxiliary Heads and OHEM) are not merely optional but essential for stabilizing SSM training, offering a new guideline for future Mamba-based research.

Limitations: While AutoMamba demonstrates significant efficiency advantages and effectively addresses the quadratic bottleneck of Transformers, we acknowledge two primary limitations. First, to rigorously isolate the architectural contributions of our Hybrid-SSM, the models were evaluated strictly under a training-from-scratch protocol. While this ensures a fair controlled baseline, it implies that the upper bound of AutoMamba’s representational capacity remains unexplored. The current empirical results do not reflect the asymptotic performance limits that could be unleashed through massive pre-training paradigms (e.g., ImageNet-22K or large-scale self-supervised learning). Consequently, the full scaling potential of our architecture in data-abundant regimes has yet to be fully realized. Second, while the theoretical linear complexity drastically reduces FLOPs, the actual inference latency of SSMs relies heavily on nascent hardware-aware parallel scan kernels, which are currently less optimized on resource-constrained edge computing platforms compared to the mature TensorRT ecosystems for CNNs.

Future Work: Looking ahead, we aim to extend the linear complexity advantage of AutoMamba to Bird’s Eye View (BEV) perception. Given that BEV transformation typically involves processing massive sequences from multi-camera inputs, the efficiency of our Hybrid-SSM is particularly well-suited for such high-token scenarios. We plan to explore multi-modal fusion architectures (integrating LiDAR and camera data) driven by Mamba to achieve robust, unified 3D scene understanding. Additionally, we will verify the real-world inference efficiency of these models on embedded autonomous driving platforms (e.g., NVIDIA Jetson Orin).

## Figures and Tables

**Table 1:** Comparative summary of existing dense prediction paradigms and their structural characteristics in the context of autonomous driving. This table highlights the inherent trade-offs and existing research gaps among current mainstream architectures. While CNNs are highly efficient on edge devices, they are inherently limited in capturing global context. Conversely, Vision Transformers (ViTs) excel at global modeling but suffer from prohibitive quadratic complexity $O\left(N^{2}\right)$ at high resolutions. Recent State Space Model (SSM) adaptations successfully achieve linear complexity $O\left(N\right)$; however, Pure SSMs often struggle to preserve local high-frequency details (e.g., small objects), while existing Hybrid SSMs compromise edge-device efficiency and incur computational redundancy due to heavy multi-branch gating mechanisms and rigid, uniform scanning strategies across all network stages. Note: N/A stands for not applicable.

Method Paradigm	Complexity	Global Context	Local Prior (Small Objects)	Scanning Strategy	Edge-Device Efficiency
**CNNs** (e.g., DeepLab)	$O\left(N\right)$	Limited	Strong	N/A	High
**ViTs** (e.g., SegFormer)	$O\left(N^{2}\right)$	Strong	Weak	N/A	Low (at high-res)
**Pure SSMs** (e.g., VMamba)	$O\left(N\right)$	Strong	Weak	Uniform (Redundant)	Medium
**Hybrid SSMs** (e.g., SegMamba)	$O\left(N\right)$	Strong	Strong (but heavy)	Uniform/Fixed	Low (Memory-bound)

**Table 2:** Comparison with SegFormer on Cityscapes and BDD100K. AutoMamba consistently outperforms SegFormer in both accuracy (mIoU) and inference speed (FPS). Note: Results are reported with Test Time Augmentation (TTA) for B0 variants. The arrows (↑) and (↓) indicate that higher and lower values are better, respectively.

Method	Backbone	Cityscapes (1024 × 1024)				BDD100K (720 × 1280)			
		mIoU ↑ (%)	mAcc ↑ (%)	aAcc ↑ (%)	FPS ↑	mIoU ↑ (%)	aAcc ↑ (%)	mAcc ↑ (%)	FPS ↑
**Segformer-B0(TTA)**	MiT-B0	66.55	74.11	94.55	25.31	44.86	91.09	50.82	62.73
**AutoMamba-B0(TTA)**	Ours	67.79	75.3	94.59	34.85	46.86	91.5	52.8	64.86
**Segformer-B2**	MiT-B2	67.82	77.36	94.49	12.54	-	-	-	-
**AutoMamba-B2**	Ours	70.17	78.93	94.69	15.43	-	-	-	-

**Table 3:** Per-class IoU comparison on the Cityscapes validation set. AutoMamba shows significant improvements in classes with vertical structures (e.g., Poles) and complex dynamic objects (e.g., Riders, Trucks).

Class	Segformer-B0	AutoMamba-B0	Segformer-B2	AutoMamba-B2
**Road**	97.46	97.4	97.19	97.31
**Sidewalk**	79.67	79.78	78.84	79.59
**Building**	89.48	89.62	89.84	90.28
**Wall**	55.39	48.67	52.6	50.45
**Fence**	44.65	45.76	47.27	49.42
**Pole**	49.99	52.77	52.43	54.36
**Traffic light**	52.31	49.57	52.63	53.19
**Traffic sign**	64.1	61.92	63.74	64.51
**Vegetation**	91.19	90.68	90.83	90.62
**Terrain**	60.91	59.19	59.37	58.6
**Sky**	94.31	94.1	94.31	94.13
**Person**	71.31	72.73	70.71	72.59
**Rider**	41.91	49.47	44.52	51.53
**Car**	91.39	92.08	92.06	92.32
**Truck**	55.23	68.01	60.94	70.58
**Bus**	64.08	68.89	69.72	79.16
**Train**	54.37	54.81	58.35	70.54
**Motorcycle**	38.8	44.93	46.94	46.75
**Bicycle**	67.92	67.65	66.36	67.24

**Table 4:** Analysis of Computational Complexity (GFLOPs) across different input resolutions. Note that SegFormer-B2 suffers from Out-Of-Memory (OOM) errors at 2048×2048, while AutoMamba maintains linear scalability. Note: The (↓) indicate that lower values are better.

Method	Backbone	Params ↓	Gflops ↓			
			512 × 512	1024 × 1024	1024 × 2048	2048 × 2048
**AutoMamba-B0**	Ours	3.918	7.614	30.455	60.909	124.928
**Segformer-B0**	MiT-B0	3.720	7.956	44.307	124.928	386.048
**AutoMamba-B2**	Ours	24.877	26.268	107.52	215.04	430.08
**Segformer-B2**	MiT-B2	24.728	25.317	151.552	431.104	OOM

**Table 5:** Impact of Auxiliary Head & OHEM (80 K Iterations). Note: The (↑) indicate that higher values are better. ✓ and × denote the use and non-use of Aux and OHEM.

Method	Aux & OHEM	mIoU ↑ (%)	mAcc ↑ (%)	aAcc ↑ (%)	ΔmIoU (%)
**SegFormer-B0**	×	59.53	68.08	93.34	-
**SegFormer-B0**	✓	58.16	67.19	92.95	−1.37
**AutoMamba-B0**	×	58.65	67.92	92.84	-
**AutoMamba-B0**	✓	59.62	68.76	92.97	+0.97

**Table 6:** Component-wise Ablation Study (80 K Iterations). Note: The arrows (↑) and (↓) indicate that higher and lower values are better, respectively. ✓ and × denote the use and non-use of Mixed Scan or DWConv.

Configuration	Mixed Scan	DWConv	Params ↓	Gflops ↓	mIoU ↑ (%)	aAcc ↑ (%)
**Pure (Baseline)**	×	×	3.908 M	30.381	49.19	91.84
**+Mixed Scan**	✓	×	3.908 M	30.381	57.74	92.63
**+DWConv**	×	✓	3.918 M	30.455	55.18	92.59
**AutoMamba (All)**	✓	✓	3.918 M	30.455	58.65	92.84

## Data Availability

The data presented in this study are openly available. The source code and pre-trained models developed in this research are publicly accessible on GitHub at https://github.com/Josue419/AutoMamba (accessed on 30 March 2026). The Cityscapes and BDD100K datasets analyzed during this study are publicly available from their respective official repositories.
